# Association between the expression of epithelial–mesenchymal transition (EMT)-related markers and oncologic outcomes of colorectal cancer

**DOI:** 10.1007/s13304-024-01865-9

**Published:** 2024-05-18

**Authors:** Mona Hany Emile, Sameh Hany Emile, Amr Awad El-Karef, Mohamed Awad Ebrahim, Ibrahim Eldosoky Mohammed, Dina Abdallah Ibrahim

**Affiliations:** 1 Pathology Department, Faculty of Medicine, Mansoura University, Mansoura, Egypt; 2 Colorectal Surgery Unit, General Surgery Department, Mansoura University Hospitals, Mansoura University, 60 El-Gomhouria Street, Mansoura, 35516 Dakahlia Egypt; 3 Medical Oncology Department, Faculty of Medicine, Mansoura University, Mansoura, Egypt

**Keywords:** Abnormal expression, Epithelial–mesenchymal transition (EMT), Markers, Oncologic outcomes, Colorectal cancer

## Abstract

**Background:**

Epithelial–mesenchymal transition (EMT) is a key step in the development of colorectal cancer (CRC) that confers metastatic capabilities to cancer cells. The present study aimed to assess the immunohistochemical (IHC) expression and impact of EMT markers, including E-cadherin, Vimentin, β-catenin, and SMAD4, on the oncologic outcomes of CRC.

**Methods:**

This was a retrospective review of 118 CRC patients. Tissue slides were retrieved from the slide archive and five tissue microarray construction blocks were constructed. IHC for E-cadherin, Vimentin, β-catenin, and SMAD4 was done. The main outcome was the association between abnormal marker expression and overall survival (OS), and disease-free survival (DFS).

**Results:**

Adenocarcinomas accounted for 71.2% of tumors, whereas 25.4% and 3.4% were mucinous and signet ring cell carcinomas. The rates of lymphovascular invasion and perineural invasion were 72.9% and 20.3%, respectively. There was a positive, significant correlation, and association between the four markers. Abnormal expression of E-cadherin was associated with significantly lower OS (*p* < 0.0001) and similar DFS (*p* = 0.06). Abnormal Vimentin expression was associated with a significantly higher rate of distant metastasis (*p* = 0.005) and significantly lower OS and DFS (*p* < 0.0001). Abnormal expression of β-catenin was associated with significantly lower OS (*p* < 0.0001) and similar DFS (*p* = 0.15). Abnormal expression of SMAD4 was associated with significantly lower OS and DFS (*p* < 0.0001). Abnormal expression of all four markers was associated with a higher disease recurrence, lower OS, and lower DFS.

**Conclusion:**

Abnormal expression of each marker was associated with lower OS, whereas abnormal expression of Vimentin and SMAD4 only was associated with lower DFS.

**Supplementary Information:**

The online version contains supplementary material available at 10.1007/s13304-024-01865-9.

## Introduction

Colorectal cancer (CRC) arises through various molecular pathways, influenced by several environmental and genetic risk factors [1, 2]. It is imperative to understand the molecular mechanisms underlying the genetic and epigenetic alterations that promote CRC progression to develop effective treatment strategies and overcome resistance to chemotherapy [3].

Epithelial–mesenchymal transition (EMT) is a complex biologic process that has been recognized as key of carcinogenesis. EMT confers metastatic capabilities to cancer cells by enhancing motility and invasion and acquiring stem cell traits. EMT also provides cancer cells with marked resistance to therapy [4]*.* EMT is accompanied by downregulation of epithelial intercellular adhesion molecules, such as epithelial–cadherin (E-cadherin), together with the acquisition of mesenchymal markers such as Vimentin [5].

A coordination of a complex network comprising molecular signaling cascades and regulators is required for EMT. EMT can be triggered by several signaling pathways, including wingless-type mouse mammary tumor virus integration site family (Wnt) and transforming growth factor-β (TGF-β) [6]. There are three different Wnt signaling pathways, the most important of which is the classic pathway in which the central mediator is β-catenin. On the other hand, the key effector of the TGF-β signaling pathway is Small Mother Against Decapentaplegic (SMAD4) which is a tumor suppressor gene that is mutated in nearly 20% of CRCs [7].

EMT is closely related to the progression of cancer and is considered a key step for local and distant recurrence. Since metastasis is the leading cause of cancer-related mortality, prevention and treatment of cancer metastases are crucial to improve outcomes. Targeting EMT pathways and their regulators can be a viable approach of cancer therapy. As a result, assessment of the role of EMT in the development of metastasis remains an important area of research [6]. The present study aimed to investigate the immunohistochemical (IHC) expression of EMT markers, including E-cadherin, Vimentin, β-catenin, and SMAD4, and their association with the oncologic outcomes of CRC.

## Patients and methods

### Study design and setting

This was a retrospective cohort study on patients with CRC who underwent resection in the period of 2012–2016. The specimens were obtained from the pathology laboratory of Gastroenterology and Oncology Centers of our University. Ethical approval for the study was obtained from the institutional review board (IRB) of our institution (MDP.18.08.10). The study was performed in accordance with the Declaration of Helsinki and is reported in adherence to the STROBE guideline of reporting observational studies. Informed consent was waived by the IRB owing to the retrospective nature of the study and the de-identified nature of data.

### Selection criteria

Patients with a confirmed diagnosis of primary CRC who underwent radical resection were included. We excluded patients with incomplete clinicopathological data, those with other malignancies, those with no available slides or blocks in the archives, specimens with repetitive tissue loss during the antigen retrieval procedure, and patients with inflammatory bowel disease (IBD).

### Evaluation of clinical and histopathological parameters

The clinicopathological data of the patients were extracted from the medical records and included age, sex, tumor size, tumor location, local recurrence, and distant metastasis. Data on follow-up and outcomes were obtained by reviewing patients’ records in the study period. Hematoxylin and eosin (H & E)-stained slides were retrieved from the slide archive and were reviewed to confirm the diagnosis of CRC. The following histopathological features were recorded for each case: histological subtype, lymphovascular and perineural invasion, neuroendocrine differentiation, extent of tumor invasion, and lymph node metastasis. Adenocarcinomas were graded according to latest WHO classification into low (GI and II) and high grades (GIII). Each tumor was assigned a stage according to the latest American Joint Committee on Cancer (AJCC) TNM staging criteria.

### Tissue microarray (TMA) construction 

Five TMA blocks were constructed using mechanical tip pencil method [8]. Empty prepared recipient paraffin blocks were prepared. A mechanical pencil tip with a thickness of 0.7 mm was used to punch out wax cylinders from the recipient block, generating holes with a diameter of about 0.8 mm. All H&E slides were reviewed and marked at the most relevant representative areas before being placed over the surface of donor paraffin blocks. Regions of paraffin blocks that correspond to the marked areas were identified and punched out with a mechanical pencil tip of 0.9 mm diameter. Tissue cores were carefully pressed out of the pencil tip with a small metal needle and transferred to recipient block holes. The cores were then inserted into recipient blocks according to a designed map for each block. Three tissue cores were punched from three different sites from each donor block of resected non-mucinous CRC, and at least six cores were taken from mucinous and signet ring CRC cases. Various normal tissue cores including normal colonic mucosa, pancreas, liver, skin, and tonsil were used as a control to the TMA technique. All fallen cores were repeated in a separate block.

### Interpretation of immunohistochemical staining results

**Fig. 1:**
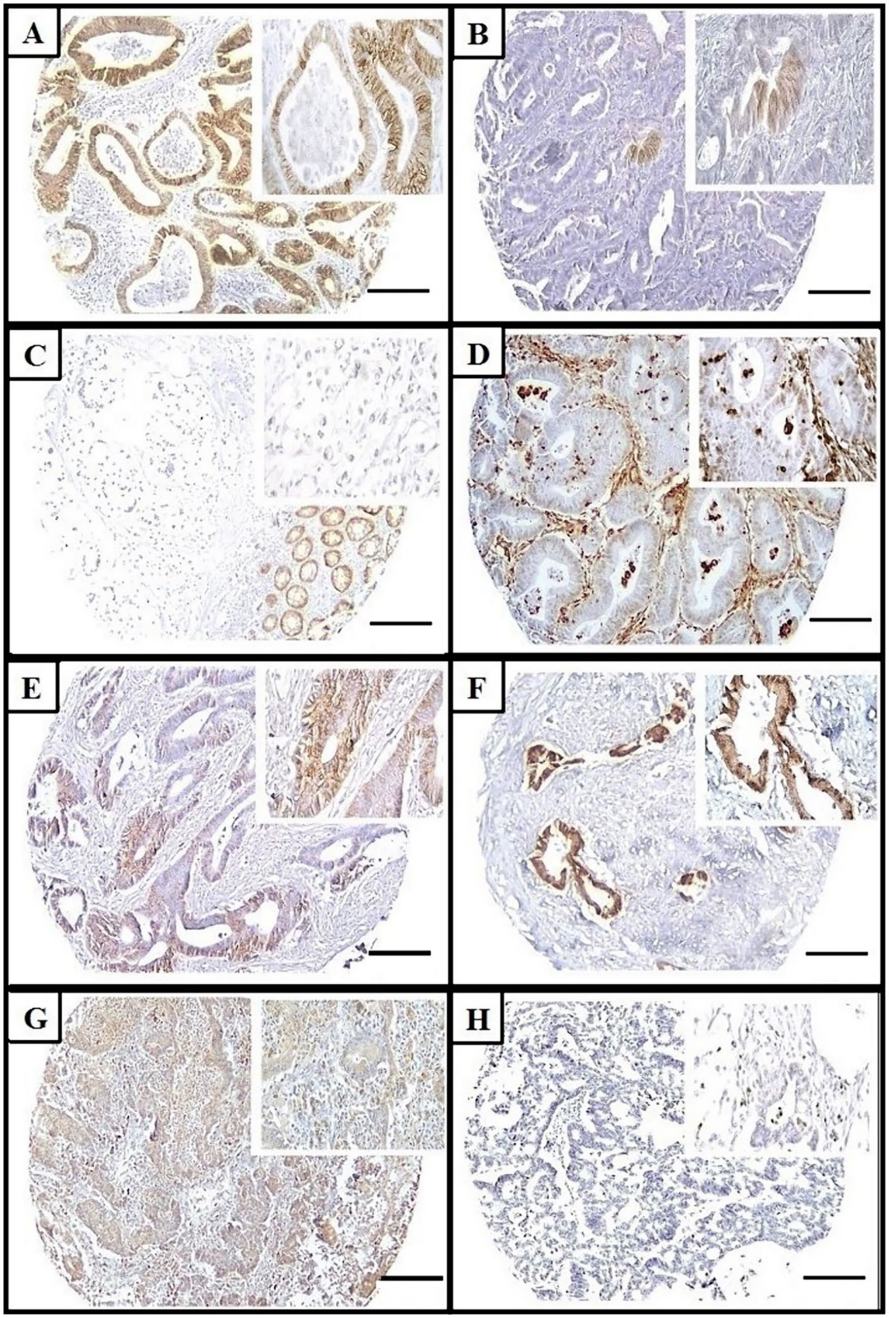
Abnormal immunohistochemical expression patterns of markers (× 100; inset × 400), scale bar = 150 µm. **A** Patchy membranous immunoreactivity in 10–90% of tumor cells for E-cadherin. **B** < 10% positive tumor cells for E-cadherin. **C** Signet ring cell carcinoma showing complete absence of staining for E-cadherin while membranous reaction of normal mucosa is positive control. **D** Positive Vimentin expression in nuclei of tumor cells. **E** Reduced membranous β-catenin staining reaction in < 70% of tumor cells. **F** Cytoplasmic and nuclear staining reaction of neoplastic epithelial cells for β-catenin. **G** Weak positive cytoplasmic SMAD4 expression in tumor cells. **H** Loss of SMAD4 expression in tumor cells while preserved staining reaction in stromal cells is an internal positive control

The methods used for immunohistochemistry and interpretation of the IHC expression of the four markers are detailed in the Supplementary Material [10–18] (Fig. 1).

### Study outcomes

The outcomes of this study were the rates of abnormal expression of E-cadherin, SMAD4, Vimentin, and β-catenin; the correlation and association between the four markers, and the association between the abnormal expression of each marker with disease recurrence, overall survival (OS), and disease-free survival (DFS). The OS was calculated from initial surgical resection to death or last contact and DFS was calculated from the operation time until the first occurrence of disease recurrence or metastasis.

### Sample size calculation

The sample size for the study was calculated using online sample size calculation software (https://clincalc.com/stats/samplesize.aspx). Considering patients survival as the primary outcome of the study and in light of previous studies [7, 19–22], we assumed four different effect sizes for OS, one for each marker (26% for E-cadherin, 30% for SMAD4, 33% for Vimentin, and 45% for β-catenin). To obtain an adequate study power of 80% that is able to detect significant difference in OS between patients with normal and abnormal expression of each marker, we selected the smallest effect size of 26%. Therefore, a minimum sample size of 112 patients was required to be included to the study.

### Statistical analysis

Data were collected, tabulated, coded then analyzed using IBM SPSS for Windows, Version 23.0. Armonk, NY. Qualitative data were described as numbers and proportions and quantitative data as median and range for non-parametric data and mean with standard deviation (SD) for parametric data after testing normality using Kolmogorov–Smirnov test. For analysis of categorical data, Chi-square test or Fischer exact test was used. For continuous data, Student *t* test was used to compare two independent groups. Spearman correlation test was used to calculate the correlation coefficient (*r*) and *p* value of correlations between different markers. The prognostic performance of each marker to predict disease recurrence was expressed as sensitivity, specificity, and accuracy. OS and DFS were calculated using Kaplan–Meier test and the Log-rank test was used to estimate statistical significance of survival differences between two groups. Differences were considered significant if *p* value was less than 0.05.

## Results

### Patient characteristics

**Fig. 2:**
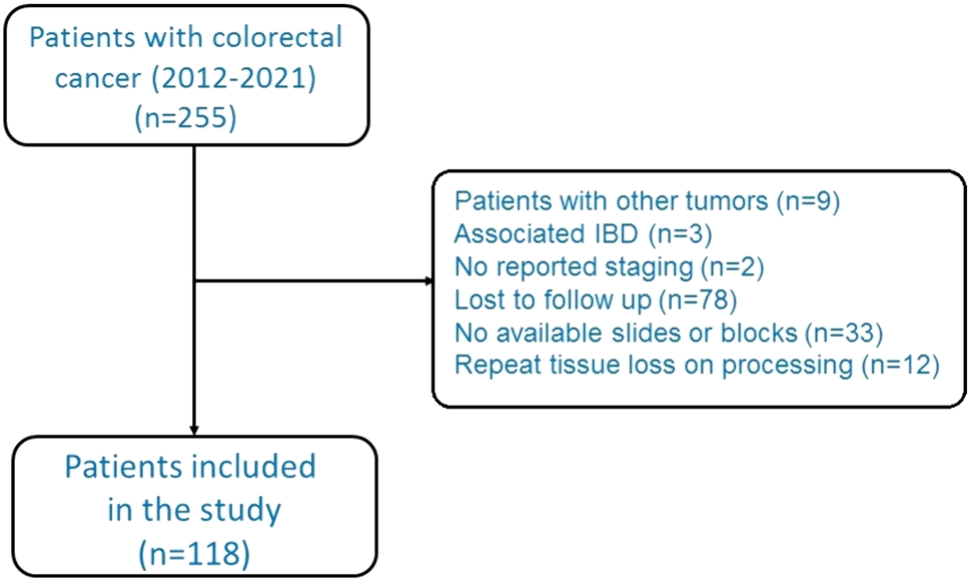
Flowchart for patient selection to the study

After screening the records of 255 consecutive patients, we excluded 9 patients with tumors other than CRC, 3 with associated IBD, 2 with no reported staging, 78 who were lost to follow-up, 33 with no available slides or blocks in archive, and 12 who showed repeated tissue loss on processing. The process of patient selection to the study is illustrated in Fig. 2. Thus, this study included 118 patients comprising 62 (52.5%) female and 56 (47.5%) male with a mean age of 55.7 ± 12.3 years.

### Tumor characteristics

#### Location and size

Forty-four (37.3%) tumors were located in the right side of the colon, forty-four (37.3%) in the left side, and thirty (25.4%) in the rectum. Twenty-one (17.8%) tumors were ≤ 4 cm in size and eighty-five (72%) were > 4 cm. Twelve tumors were received as pieces, or presented as infiltrating circumferential masses and thus had no recorded size.

#### Histology and high-risk features

There were 84 (71.2%) adenocarcinomas (8.3% grade I, 85.7% grade II, and 6% grade III), 30 (25.4%) mucinous carcinomas, and 4 (3.4%) signet ring cell carcinomas. Twenty-nine (34.5%) adenocarcinomas had mucoid activity and nine (30%) mucinous adenocarcinomas had a signet ring component in < 50% of cells. Regarding high-risk features, 86 (72.9%) tumors had lymphovascular invasion and 24 (20.3%) had perineural invasion. Neuroendocrine differentiation was detected in six (5%) tumors. Thirty-one (26.3%) tumors were on top of pre-existing adenomas and associated schistosomiasis was found in six (5%) patients.

#### Stage

Eighteen (15.3%) tumors were of T2 stage, seventy-four (62.7%) of T3 stage, twenty-six (22%) of T4 (eight of T4a and eighteen of T4b). Fifty-nine (50%) tumors were of N0 stage, thirty (25.4%) of N1 stage, and twenty-nine (24.6%) of N2. Eighty-six (72.9%) tumors were of M0 stage and thirty-two (27.1%) of M1 stage. Nine (7.6%) tumors were of TNM stage I, thirty-nine (33.1%) of stage II, thirty-eight (32.2%) of stage III, and thirty-two (27.1%) of stage IV.

### Treatment and outcomes

All patients were given adjuvant therapy, 22% of whom received concurrent radiotherapy that was palliative in four patients. Local recurrence was detected in 24 (20.3%) patients and distant metastasis in 32 (27.1%) patients. The 5-year OS rate was 58.5% and the 5-year DFS was 53.1%. The median time to recurrence and metastasis was 23.5 (range 6–94) months.

### Immunohistochemical markers status

**Fig. 3:**
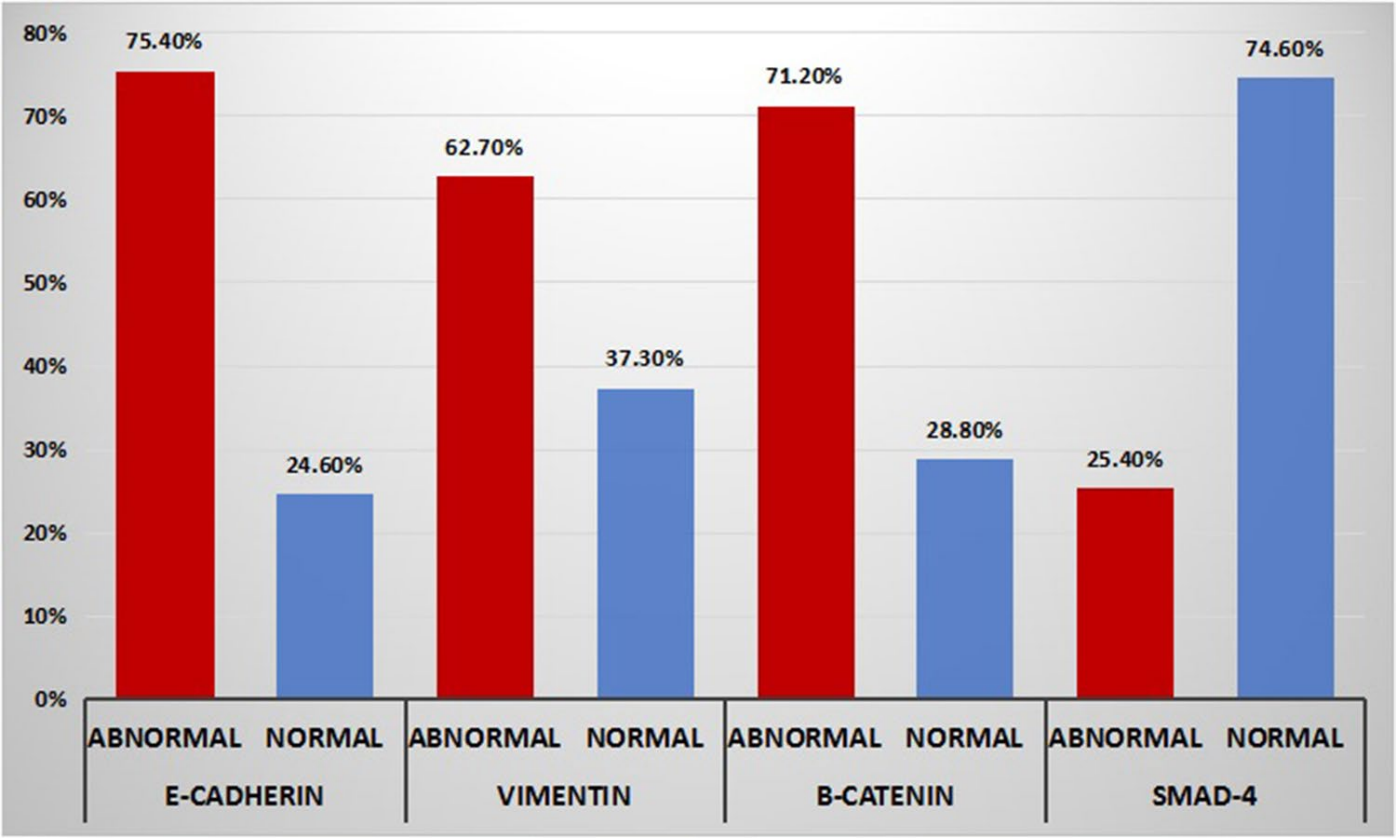
Rates of abnormal expression of the four markers in immunohistochemistry

The IHC status of CRC patients with normal or abnormal expression of the four markers is shown in Fig. 3. Overall, 89 (75.4%) patients had abnormal expression of E-cadherin (62 aberrant expression and 27 negative); 74 (62.7%) had abnormal (positive) Vimentin expression; 84 (71.2%) had abnormal expression for β-catenin (reduced membranous staining in 11 or ectopic expression in the cytoplasm/nucleus in 73); and 30 (25.4%) had abnormal (low) SMAD4 expression.

### Correlation and association between the four markers

There was a strong, positive, significant correlation between E-cadherin and Vimentin (*r* = 0.62, *p* < 0.00001) and between E-cadherin and β-catenin (*r* = 0.76, *p* < 0.0001). The correlation between Vimentin and β-catenin was moderate, positive, and significant (*r* = 0.5, *p* < 0.0001). There were positive, weak, and significant correlations between SMAD4 and E-cadherin (*r* = 0.33, *p* = 0.0002), SMAD4 and Vimentin (*r* = 0.32, *p* = 0.0003), and between SMAD4 and β-catenin (*r* = 0.28, *p* = 0.001).

There were highly significant (*p* < 0.0001) associations between the abnormal expression of E-cadherin and Vimentin, E-cadherin and β-catenin, Vimentin and β-catenin, SMAD4 and E-cadherin, and a significant association between SMAD4 and Vimentin (*p* = 0.0003), and SMAD4 and β-catenin (*p* = 0.001) as shown in Table 1**.**

**Table 1:** Association between the abnormal expression of the four markers

Abnormal expression	Vimentin (*n* = 74)	β-Catenin (*n* = 84)	E-cadherin (*n* = 89)	SMAD4 (*n* = 30)
Vimentin (*n* = 74)		66 (78.5%) *	71 (79.8%)*	27 (90%)**
β-Catenin (*n* = 84)	66 (89.2%)*		81 (91%)*	28 (93.3%)^#^
E-cadherin (*n* = 89)	71 (95.9%)*	81 (96.4%)*		30 (100%)*
SMAD4 (*n* = 30)	27 (36.5%)**	28 (33.3%)^#^	30 (33.7%)*	

^*^p < 0.0001, **p = 0.0003, #*p* = 0.001

### Association between abnormal marker expression and oncologic outcomes

**Fig. 4:**
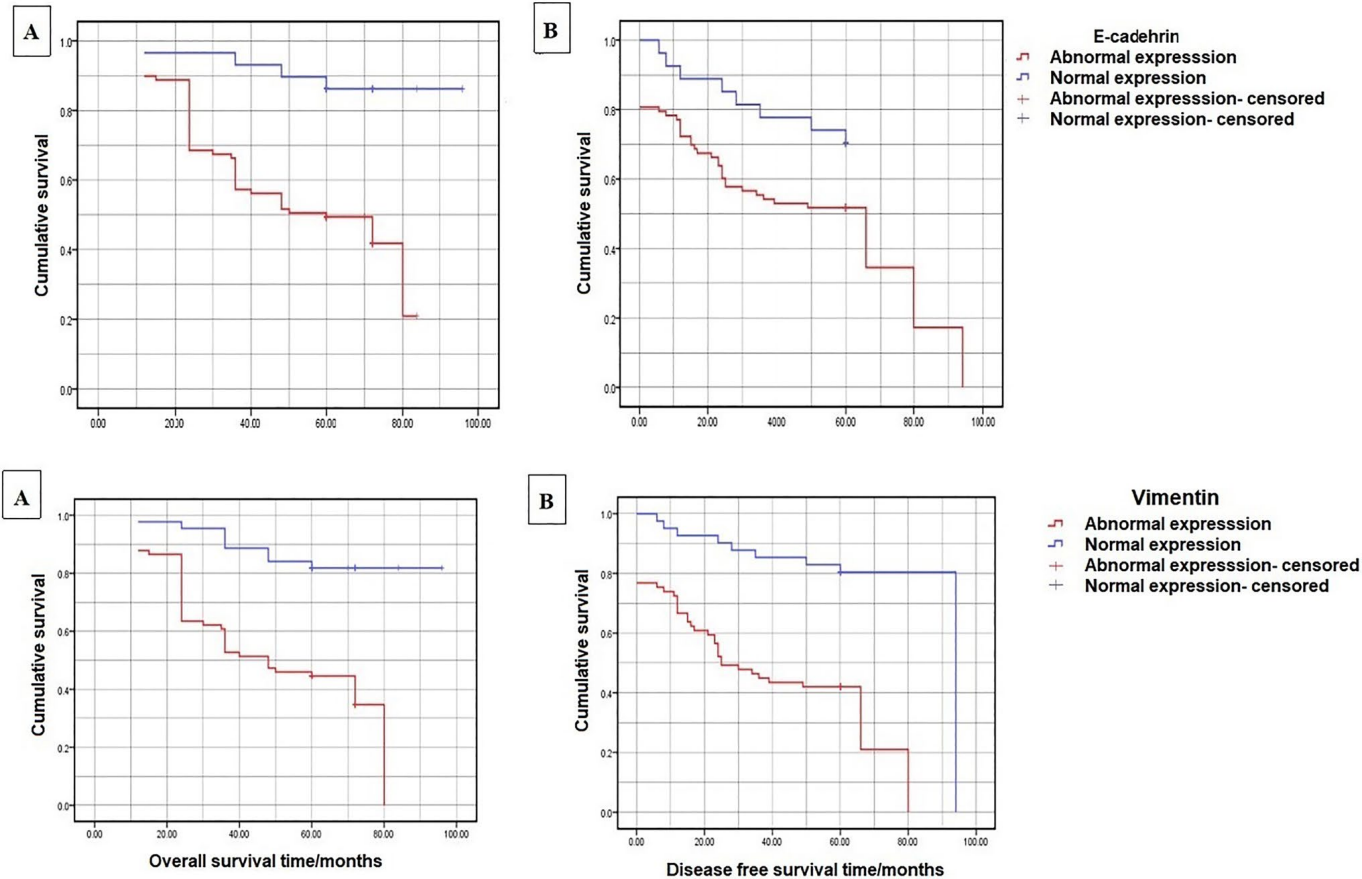
Overall and disease-free survival according to expression of E-cadherin and Vimentin

Abnormal expression of *E-cadherin* was associated with a higher disease recurrence (52.8% vs 31%) than normal expression, yet this difference was not statistically significant (Table 2). According to Kaplan–Meier statistics, patients with abnormal expression of E-cadherin had significantly lower OS (53.8% vs 88.1%; *p* < 0.0001) and similar DFS (47.6% vs 50.5%; *p* = 0.06) to patients with normal expression (Fig. 4).

**Table 2:** Association between abnormal marker expression and disease recurrence

Variable	Local recurrence	Distant metastasis	Overall recurrence
E-cadherin			
Abnormal expression (*n* = 89)	20	27	47
Normal expression (= 29)	4	5	9
*P* value	0.43	0.12	0.06
Vimentin			
Abnormal expression (*n* = 74)	19	27	46
Normal expression (*n* = 44)	19	5	24
*P* value	0.08	**0.005**	0.53
β-Catenin			
Abnormal expression (*n* = 84)	20	24	44
Normal expression (*n* = 34)	4	8	12
*P* value	0.2	0.74	0.14
SMAD4			
Abnormal expression (*n* = 30)	10	13	23
Normal expression (*n* = 88)	14	19	33
*P* value	0.07	**0.04**	**0.0005**

Bold values reflect significant p values <0.05

Abnormal *Vimentin* expression was associated with a significantly higher rate of distant metastasis (*p* = 0.005) (Table 2) and higher odds of overall recurrence than patients with normal expression (OR = 5.58, 95% CI 2.39–13.04, *p* = 0.0001). Patients with abnormal expression of Vimentin had significantly lower OS (49.8% vs 85.5%; *p* < 0.0001) and lower DFS (38.1%vs 81.8%; *p* < 0.0001) than patients with normal expression (Fig. 4).

**Fig. 5:**
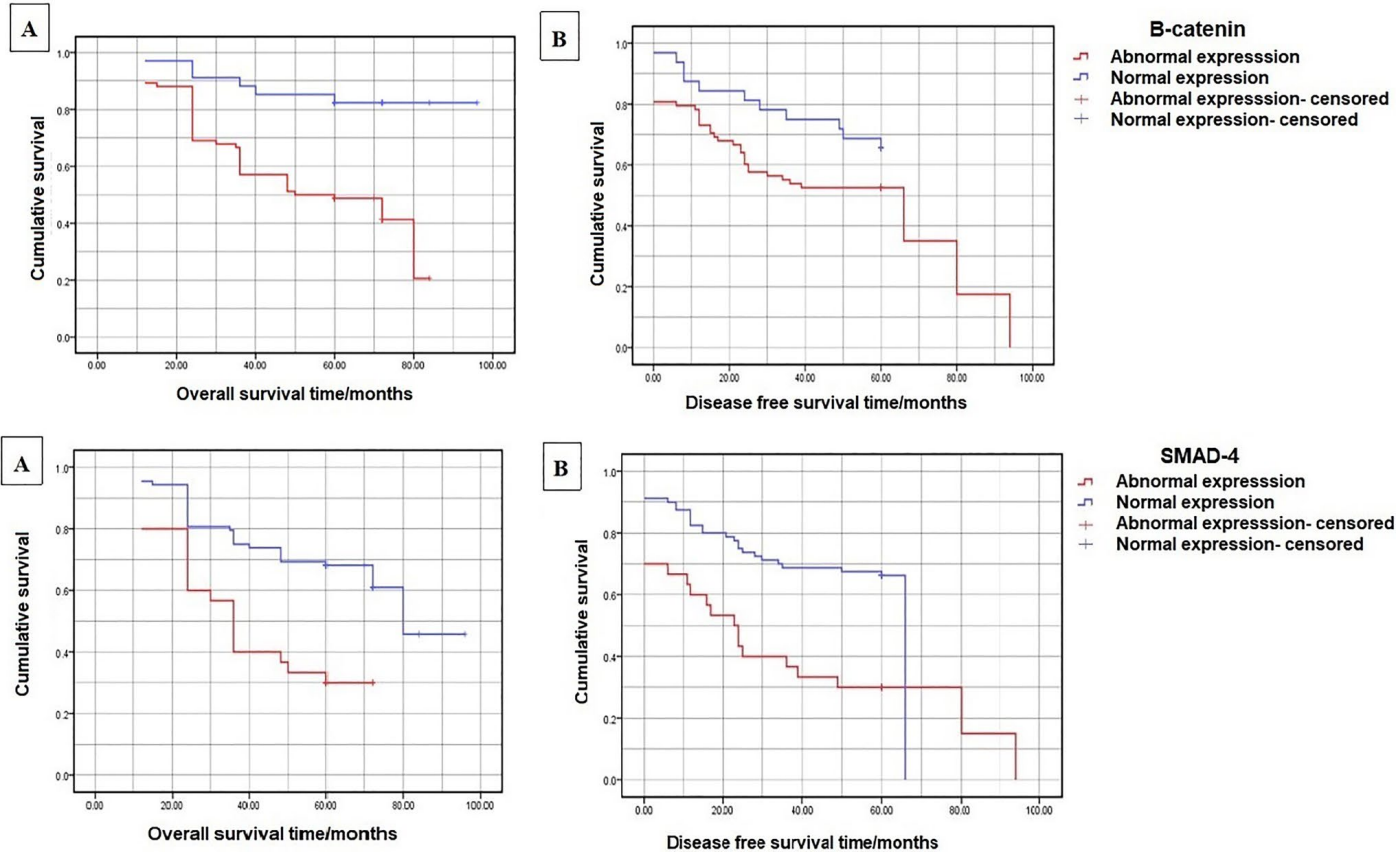
Overall and disease-free survival according to expression of β-catenin and SMAD4

The difference in the recurrence rates between abnormal and normal expression of *β-catenin* was not statistically significant (*p* = 0.14) (Table 2). Patients with abnormal expression of β-catenin had significantly lower OS (53.6% vs 84.8%; *p* < 0.0001) yet a similar DFS (47.8% vs 48.1%, *p* = 0.15) compared to patients with normal expression (Fig. 5).

Abnormal (low) expression of *SMAD4* was associated with a significantly higher rate of distant metastasis (*p* = 0.04) and overall recurrence (*p* = 0.0005) (Table 2) and significantly higher odds of overall recurrence (OR = 5.47, 95% CI 2.11–14.15, *p* = 0.0004). Patients with low expression of SMAD4 had significantly lower OS (41.1% vs 70.6%; *p* < 0.0001) and lower DFS (35.5% vs 49.4%; *p* < 0.0001) than patients with high expression (Fig. 5).

Seventy-one patients had abnormal expression of E-cadherin and vimentin, the two canonical markers for E and M phenotypes, whereas twenty-six had normal expression of both markers. Abnormal expression of E-cadherin and vimentin was associated with lower OS (38% vs 84.6%, *p* < 0.0001) and DFS (35.2% vs 65.4%, *p* = 0.011) than normal expression of both markers. Twenty-five patients had abnormal expression of the four markers and twenty-three had normal expression of the four markers. Abnormal expression of the four markers was associated with a significantly higher disease recurrence (80% vs 39.1%; *p* = 0.009), lower OS (24% vs 86.9%; *p* < 0.0001), and lower DFS (20% vs 60.9%; *p* = 0.009) as compared to patients with normal expression of the four markers.

### Prognostic accuracy of the EMT-related markers

Abnormal expression of E-cadherin had the highest sensitivity in prediction of disease recurrence (83.9%) whereas abnormal SMAD4 expression had the highest specificity (88.7%) and abnormal expression of the four markers together had the highest accuracy. A summary of the prognostic accuracy of the four markers is shown in Table 3.

**Table 3:** Prognostic accuracy of the EMT-related markers in CRC

Marker	Sensitivity (%)	Specificity (%)	Accuracy (%)	Positive predictive value (%)	Negative predictive value (%)
E-cadherin	83.9	32.3	56.8	52.8	68.9
Vimentin	82.1	54.8	67.8	62.2	77.3
β-Catenin	78.6	35.5	55.9	52.4	64.7
SMAD4	41.07	88.7	66.1	76.7	62.5
All markers	68.9	73.7	70.8	80	60.9

## Discussion

EMT is thought to be a critical mechanism of tumor progression and metastasis. Assessment of EMT markers in primary CRC can improve risk stratification and treatment decisions by identifying patients who are at a potentially higher risk for metastatic disease [23]. Targeting EMT has emerged as a novel strategy against tumor progression and metastatic dissemination, as well as against resistance to chemotherapy and radiotherapy [24]. The present study assessed the expression of the EMT-related markers and the association between their abnormal expression and disease recurrence and survival. Around 75% of tumors had abnormal expression of E-cadherin, in line with Reinacher-Schick et al. [18] who reported normal expression patterns of E-cadherin in only 25.5% of tumors. However, another study [6] reported a lower rate of abnormal E-cadherin expression in 46% of cases. This difference may be attributable to different assessment methods of E-cadherin expression, including a lower cut-off value of preserved normal membranous staining of E-cadherin.

According to our study, 62.7% of tumors showed positive Vimentin staining. This was slightly higher than that reported by other studies [25, 26] (56%). However, much lower rates were reported by other investigators [27, 28]. The wide variation in the detection rate of Vimentin expression might be attributed to the use of different cut-off values, ranging from 5 to 10%, in the studies. Beta-catenin is a fundamental structural component of cadherin-based adherens junctions that form an important membrane complex with E-cadherin [29]. In the present study, 71.2% of tumors had abnormal expression for β-catenin, compared to 57–63% in other studies [18, 30]. Again, the use of different methods of assessment of β-catenin expression might be the cause of such variations.

SMAD4 is regarded as the central mediator of TGF-β signaling in epithelial cell [31]. In our study, 25.4% of tumors had abnormal/low expression for SMAD4 and this was lower than that reported by other studies [18, 32]. A possible explanation of the discrepancy in SMAD4 expression is the different classification systems used to evaluate the IHC expression. In addition, because of the ambiguity in defining low-level SMAD4 expression, the reported prevalence of low expression may widely vary from 2.3% to 75.2% [33].

There was a significant and positive correlation and association between the four markers. The positive correlation and association between E-cadherin, β-catenin, and SMAD4 was discussed by Müller et al. [34] and Reinacher-Schick et al. [18] who reported that retention of normal patterns of E-cadherin and β-catenin expression was correlated to normal levels of SMAD4 expression, while the lack of expression of SMAD4 was associated with a lack of expression of both E-cadherin and β-catenin. Also, the expression of E-cadherin and β-catenin seemed to be parallel with each other. The significant correlation and association between Vimentin, β-catenin, and SMAD4 was also highlighted in other studies [7, 14].

According to Niknami et al. [26], as cancer progresses, the mesenchymal markers such as Vimentin tend to abnormally increase while structural adhesion proteins such as E-cadherin tend to decrease in CRC, in concordance our results. Interestingly, we found some cases that showed both epithelial/mesenchymal phenotypes. This might be explained by the recently recognized feature of cancer cells being able to undergo partial rather than complete EMT. These partial EMT states were thought to enhance invasive capabilities, generate cancer stem cells, and promote resistance to anti-cancer drugs. However, the molecular mechanisms that regulate partial EMT are still unclear [35].

One study [36] identified mRNA expression of EMT-related cell markers including E-cadherin and Vimentin in commonly used human colorectal cancer cell lines. They found that EMT was relevant in 10 out of the 11 human CRC cell lines. These findings support the concept of high metastatic and tumorigenic potentials of these cancer cell lines. Another study [37] used a panel of HCT116 cell lines with differential β-catenin mutation status and suggested that β-catenin activation induces EMT progression by modifying E-cadherin-dependent cell–cell junctions, and thereby contributes to CRC aggressiveness. A previous study [38] highlighted that SMAD4 may play a vital role in the sensitivity of CRC cells to chemotherapeutic agents by promoting EM. It showed that a low expression of SMAD4 was present in CRC tissues analyzed by TCGA and in four CRC cell lines. They stated that silencing SMAD4 partly reversed the effects of cetuximab on the mRNA and protein expression levels of Vimentin and E-cadherin. Also, silencing SMAD4 attenuated the sensitivity of SW480 CRC cells to cetuximab-based treatment; this effect was reflected in increased cell viability and slightly increased migration and invasion.

Regarding the prognostic value of the markers, abnormal expression of E-cadherin was associated with significantly lower OS and similar DFS to normal expression whereas abnormal Vimentin expression was associated with a significantly higher recurrence rate and significantly lower OS and DFS than normal expression, in agreement with another study [13]. Furthermore, abnormal expression of β-catenin was associated with significantly lower OS than normal expression, consistent with the finding of another study [21]. Conversely, abnormal expression of β-catenin expression was not associated with significant effects on disease recurrence and DFS. This finding was in concordance with Balzi et al. [39] who found that only nuclear expression of β-catenin is significantly associated with DFS at certain pathological stages of the disease. Finally, abnormal expression of SMAD4 was associated with a significantly higher rate of recurrence and significantly lower OS and DFS, in agreement with previous studies [7, 17, 40].

Interestingly, abnormal expression of the four markers together was significantly associated with higher disease recurrence and worse survival. These results were consistent with prior research on colorectal and gastric cancers that concluded a higher impact of concurrent changes of EMT- and cancer stem cell (CSC)-related markers on the biological behavior rather than the alteration of a single EMT- or CSC-related protein [41].

Limitations of the present study include its single-center and retrospective nature. The limited sample size and small number of patients included is another main limitation to the study that may hinder conducting nuanced secondary analyses and drawing definitive conclusion. EMT was analyzed in our study as a dogmatic binary process, whereas now there is evidence on the existence of partial EMT states [42–44]. The lack of resources for genomic expression profiling precluded performing bioinformatics analysis of microarray data which is recommended in future research.

## Conclusion

Abnormal expression of E-cadherin had significantly lower OS and similar DFS survival to normal expression. Abnormal Vimentin expression was associated with a significantly higher rate of distant metastasis and significantly lower OS and DFS than normal expression. Abnormal expression of β-catenin was associated with significantly lower OS and similar DFS to patients with normal expression. Abnormal expression of SMAD4 was associated with a significantly higher rate of distant metastasis and overall recurrence with significantly lower OS and DFS than patients with normal expression. The combination analysis of all four markers showed a significant association with disease recurrence and survival.

## Supplementary Information

Below is the link to the electronic supplementary material.

Supplementary file1 (DOCX 84 KB)

## Data Availability

The data that support the findings of this study are available on request from the corresponding author. The data are not publicly available due to privacy or ethical restriction.
