# Supplementary material for: Association between the expression of epithelial–mesenchymal transition (EMT)-related markers and oncologic outcomes of colorectal cancer
Source: Updates Surg. 2024 May 18;76(6):2181–91. doi: 10.1007/s13304-024-01865-9 (PMC11541317; doi:10.1007/s13304-024-01865-9)
Supplement: Supplementary file 1 — Supplementary file1 (DOCX 84 KB) [file 13304_2024_1865_MOESM1_ESM.docx]

***Immunohistochemistry***

After trimming, 4-μm-thick sections of paraffin-embedded tissue were prepared from each TMA block. Tissue sections were placed on glass slides with positive charge and dried in an oven at 10 ^0^C for one hour.

The slides were then deparaffinized in xylene for thirty minutes before being rehydrated with graded ethanol concentrations. The next step was heat-induced epitope retrieval in a pressure cooker steamer using citrate buffer PH (6.0). Upon completion, the slides were rinsed with five changes of distilled water and phosphate-buffered saline (PBS). Endogenous peroxidase activity was blocked by immersing the slides into 3% hydrogen peroxide for ten minutes.

After that, the slides were incubated with antibodies against E-cadherin (mouse monoclonal, Ig G, ready-to-Use, Agilent Dako), Vimentin (mouse monoclonal, Ig G, ready-to-Use, Agilent Dako), β-catenin (mouse monoclonal, Ig G, ready-to-Use, Agilent Dako) and SMAD4 (rabbit polyclonal, Ig G, diluted 1:100, Biospes) for sixty minutes.

The antibodies were incubated with horseradish peroxidase (HRP) labelled polymer (UltraVision One HRP polymer, Agilent Dako, USA) for thirty minutes at room temperature, washed four times in buffer solution. Then, one drop of 3,3’-Diaminobenzidine (DAB) chromogen was added to one ml of Naphthol Phosphate substrate and mixed thoroughly. The solution was then applied to tissue section and incubated for fifteen minutes.

Tissue sections were then washed with distilled water and counterstained with hematoxylin. After that, the dehydration process was carried out using ascending grades of alcohol and xylene. Finally, Dibutylphthalate Polystyrene Xylene (DPX) mounting medium was used to mount the slides and slides were covered.

***Interpretation of Immunohistochemical staining results***

E-cadherin expression

According to Kanazawa, et al [10], expression of E-cadherin was considered as normal if >90% of tumor cells expressed uniform membranous immunoreactivity. Sections stained with <10% positive cells or with complete absence of staining were considered as negative. Heterogeneous staining with patchy or focal membranous immunoreactivity in 10-90% of cells and immunostaining with altered cytoplasmic or nuclear cellular distribution were considered aberrant. For analytical purpose, heterogeneous and aberrant/negative staining were considered as abnromal expression. Strong membranous E-cadherin expression in normal colorectal epithelial cells and epidermal keratinocytes in normal skin tissue cores were used as positive controls [11].Top of Form

Vimentin expression

According to Pino et al [12], Vimentin was normally expressed in the cytoplasm of mesenchymal cells but not in normal mucosa. If there was positive cytoplasmic, membranous, or nuclear staining in CRC epithelial cells, regardless of the number, it was classified as positive (abnormal expression). Vimentin expression in the colonic mucosal lymphocytes and stromal cells were used as internal positive control. Normal human tonsil tissue cores were also used as positive controls [13].

β-catenin expression

According to Lv et al [14], if >70% of the cancer cells showed β-catenin membrane staining, it was regarded as normal expression; otherwise, it was considered lacking of membranous staining. If >10% of the cancer cells showed cytoplasmic and/or nucleus positive staining, it was defined as ectopic expression. Reduced/lack of membranous staining and ectopic expression in the cytoplasm and nucleus were categorized as abnormal β-catenin expression. Membranous staining of β-catenin seen in the cell membranes of normal colonic epithelial cells and normal liver tissue cores were used as positive controls [15].

SMAD4 expression

According to Salovaara et al [16]*,* the expression of SMAD4 in tumor cells is interpreted using semiquantitative scale ranging from 0 to +++. Tumors displaying absence of staining or positive reaction in < 5% of tumor cells were classified as negative (0). Nuclear as well as cytoplasmic in >5% of tumor cells were considered positive. Weak staining was classified as (+). Moderate to strong staining comparable with normal tissue expression were classified as (++ to +++). For analytical purposes tumors with staining levels of 0 and + were considered as low (abnormal) SMAD4 expression, and staining levels of ++ and +++ were considered as high (normal) [17]. Normal colonic mucosa, stromal fibroblasts and lymphoid cells showing moderate to strong immunostaining for SMAD4 served as a positive internal control [18].
